# The Landé factors of electrons and holes in lead halide perovskites: universal dependence on the band gap

**DOI:** 10.1038/s41467-022-30701-0

**Published:** 2022-06-02

**Authors:** E. Kirstein, D. R. Yakovlev, M. M. Glazov, E. A. Zhukov, D. Kudlacik, I. V. Kalitukha, V. F. Sapega, G. S. Dimitriev, M. A. Semina, M. O. Nestoklon, E. L. Ivchenko, N. E. Kopteva, D. N. Dirin, O. Nazarenko, M. V. Kovalenko, A. Baumann, J. Höcker, V. Dyakonov, M. Bayer

**Affiliations:** 1 Experimentelle Physik 2, Technische Universität Dortmund, 44227 Dortmund, Germany; 2 Ioffe Institute, Russian Academy of Sciences, 194021 St. Petersburg, Russia; 3 Department of Chemistry and Applied Biosciences, Laboratory of Inorganic Chemistry, ETH Zürich, 8093 Zürich, Switzerland; 4 Department of Advanced Materials and Surfaces, Laboratory for Thin Films and Photovoltaics, Empa—Swiss Federal Laboratories for Materials Science and Technology, 8600 Dübendorf, Switzerland; 5 Experimental Physics VI, Julius-Maximilian University of Würzburg, 97074 Würzburg, Germany

**Keywords:** Semiconductors, Spintronics, Magneto-optics, Ultrafast photonics, Quantum mechanics

## Abstract

The Landé or *g*-factors of charge carriers are decisive for the spin-dependent phenomena in solids and provide also information about the underlying electronic band structure. We present a comprehensive set of experimental data for values and anisotropies of the electron and hole Landé factors in hybrid organic-inorganic (MAPbI_3_, MAPb(Br_0.5_Cl_0.5_)_3_, MAPb(Br_0.05_Cl_0.95_)_3_, FAPbBr_3_, FA_0.9_Cs_0.1_PbI_2.8_Br_0.2_, MA=methylammonium and FA=formamidinium) and all-inorganic (CsPbBr_3_) lead halide perovskites, determined by pump-probe Kerr rotation and spin-flip Raman scattering in magnetic fields up to 10 T at cryogenic temperatures. Further, we use first-principles density functional theory (DFT) calculations in combination with tight-binding and *k* ⋅ *p* approaches to calculate microscopically the Landé factors. The results demonstrate their universal dependence on the band gap energy across the different perovskite material classes, which can be summarized in a universal semi-phenomenological expression, in good agreement with experiment.

## Abstract (web summary)

The Landé factors govern all the spin-related basic phenomena and are the key parameters which guide spintronics applications. Here, Kirstein et al. demonstrate a universal dependence of the Landé factors on the bandgap energy of several perovskite materials.

## Introduction

Lead halide perovskite materials have attracted huge attention in recent years due to their exceptional electronic and optical characteristics, which make them highly promising for various applications in photovoltaics^1,2^, optoelectronics^3–8^, X-ray detectors^9,10^, etc. Their chemical formula *A*Pb*X*_3_ where the cation *A* = cesium (Cs), methylammonium (MA), formamidinium (FA) and the anion *X* = Cl, Br, I, offers a huge flexibility in composition making the band gap tunable from the infrared up to ultraviolet spectral range. Interestingly, the perovskite band structure is inverted compared to common III-V and II-VI semiconductors. As a result, the strong spin-orbit interaction influences mostly the conduction band rather than the valence band. Also strong Rashba spin splittings have been predicted both for the valence and conduction bands^11^.

Detailed studies of the band structure require a concerted effort of suitable experimental and theoretical approaches. The low charge carrier mobility^12^ hampers methods like electrical transport, also in magnetic field, and cyclotron resonance, which are usually applied to study the band structure of solids, while ion diffusion obstructs the application of capacity-based methods. Angle resolved photoemission spectroscopy^13–15^ provides promising results but, so far, with insufficient accuracy. Optics in strong magnetic fields gives access to carrier effective masses and exciton features^16,17^, where the parameter values can be, however, influenced by the field through band mixing.

Spin physics provides high precision tools for addressing the electronic states in the vicinity of the band gap. Namely, the Landé or *g*-factors of electrons and holes are inherently linked via their values and anisotropies to the band parameters, which also determine the charge carrier effective masses^18,19^. On the other hand, the Landé factors are the key parameters for the coupling of spins to a magnetic field and thus govern related basic phenomena and spintronics applications, which belong to a largely uncharted area for perovskites. The first concise reports show great promise demonstrating optical orientation^5,20–23^ and optical alignment^21^, polarized emission in magnetic field^24–26^, coherent spin dynamics^27–30^, and nuclear magnetic resonance^31–33^.

Here, we study the Landé factors of electrons and holes for representative crystals out of the class of lead halide perovskites: MAPbI_3_, MAPb(Br_0.5_Cl_0.5_)_3_, MAPb(Br_0.05_Cl_0.95_)_3_, FA_0.9_Cs_0.1_PbI_2.8_Br_0.2_, FAPbBr_3_ and CsPbBr_3_. Pump-probe Kerr rotation and spin-flip Raman scattering with ultimate resolution in the temporal and spectral domains, respectively, are used to measure the Landé factor tensor components in magnetic fields ranging from 20 mT up to 10 T. The discovered universal dependence of the *g*-factors on the band gap energy is confirmed by first-principles DFT calculations combined with tight-binding and ***k*** ⋅ ***p*** perturbation theory. Thereby we get access to the key band structure parameters and develop a reliable model to predict the Landé factors for the whole family of hybrid and inorganic lead halide perovskites, both for bulk crystals and nanostructures.

## Results

**Fig. 1:**
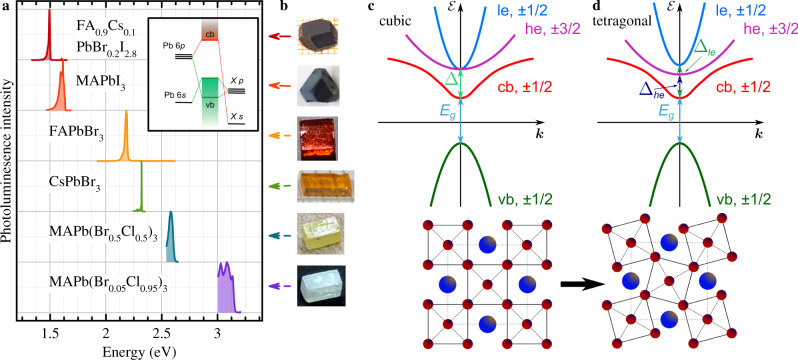
Lead halide perovskite samples. **a** Photoluminescence spectra of the crystals under study. The spectra are normalized and vertically shifted for clarity. The PL is measured at *T* = 1.6 − 10 K. Inset shows a sketch of the contribution of atomic orbitals to the electronic bands around the band gap in lead halide perovskites. **b** Photographs of representatives of the samples under study. Arrows point to the respective PL spectrum. **c**, **d** Band structure around the direct gap for the cubic and tetragonal lattices. The states at the top of the valence band (vb, holes) and the bottom of the conduction band (cb, electrons) are spin-degenerate having spin ±1/2. The degenerate heavy (he, ±3/2) and light (le, ±1/2) electron bands are split-off significantly from the cb by the spin-orbit coupling Δ. A symmetry reduction from cubic to tetragonal lifts the he-le degeneracy, leading to individual split-off energies Δ_*he*/*le*_. In the bottom panels the corresponding lattice structure is sketched with the [Pb*X*_6_]^−4^ octahedra (dark red symbols show the halogen *X* anion) and *A* the inorganic/organic cation (dark blue). An octahedra twist provides the phase transition from cubic to tetragonal symmetry for the aristotype cubic structure (dashed line square)^34,37^.

Out of the six investigated samples three representatives, namely the hybrid organic-inorganic MAPbI_3_ and FA_0.9_Cs_0.1_PbI_2.8_Br_0.2_ as well as the all-inorganic CsPbBr_3_ lead halide perovskite are chosen for discussion in detail. All samples are shown in Fig. 1b, together with photoluminescence (PL) spectra measured at the temperature of *T* = 1.6 − 10 K (Fig. 1a). The PL is contributed by recombination of bound excitons. The band gaps of the different materials vary from 1.527 eV up to 3.157 eV, resulting in the black to transparent colors of the studied crystals.

A sketch of the atom orbitals contributing to the electronic bands in the band gap vicinity is shown in the insert of Fig. 1a. The valence band (vb) is formed by hybridization of Pb *s* and halogen *X* *p* orbitals and the conduction band by Pb *p* orbitals with some hybridization with the halogen *X* *s* orbital. The band structure around the direct band gap for cubic crystal symmetry is shown in Fig. 1c, see SI for details. The valence band is simple and has spin ± 1/2. The lowest conduction band (cb) has also spin ±1/2, while the states of the heavy (he, ±3/2) and light (le, ±1/2) electrons are split from the cb by the spin orbit coupling Δ. Commonly, perovskite crystals have cubic crystal symmetry at elevated temperatures, but at lower temperatures they undergo a phase transition by octahedral tilting (see the sketch in the bottom panel of Fig. 1d) to the tetragonal phase and, with further temperature reduction, to the orthorhomic phase^34^. In a nutshell, the condensation of M and R zone-boundary phonons with decreasing temperature^35,36^ results in reconfiguration of chemical bonds such that the densest package configuration is realized^34,37^. The octahedral tilting lifts the degeneracy of the light and heavy electron states, Δ_*le*_ > Δ_*he*_, see the upper part of Fig. 1d.

The Zeeman splitting of the carrier spin states in magnetic field ***B*** is described by the Hamiltonian

1 $${{{{{{{{\mathcal{H}}}}}}}}}_{Z}=\frac{{\mu }_{B}}{2}{g}_{\alpha \beta }{\sigma }_{\alpha }{B}_{\beta },$$

where *μ*_*B*_ is the Bohr magneton, the indices *α*, *β* = *x*, *y*, *z* denote the Cartesian components, *g*_*αβ*_ are the elements of the *g*-factor tensor, and *σ*_*α*_ are the spin Pauli matrices. The *g*-factor anisotropy can reveal information on the crystal symmetry (orthorhombic in our case), while the magnitudes of *g*_*αβ*_ are directly linked to the key band structure parameters: the band gaps and the interband momentum matrix elements^18,38^.

Experimentally, the *g*-factor can be evaluated from the measured Zeeman splitting *E*_*Z*_ by means of

2 $${E}_{Z}=g{\mu }_{B}B.$$

The Zeeman splitting can be measured by various techniques. In the spectral domain, spin-flip Raman scattering (SFRS) provides the required high resolution^39^. Here, *E*_*Z*_ is equal to the Raman shift from the exciting laser line (Methods). In the temporal domain, time-resolved pump-probe Kerr rotation (TRKR)^40^ can give access to the coherent spin dynamics of carriers and therefore, the Larmor precession frequency in a transverse magnetic field, which is linked to the *g*-factor via

3 $${\omega }_{{{{{{{{\rm{L}}}}}}}}}=\frac{{\mu }_{B}}{\hslash }gB.$$

Both techniques are well established in experiments addressing the spin physics in semiconductors^18,41^. SFRS is applicable only in strong magnetic fields exceeding a few Tesla to obtain a sufficiently large shift from the laser for detection, but is to be preferable for identification of the involved electronic states and mechanisms via polarization analysis, while TRKR has a high precision even in weak magnetic fields and gives access to the spin dynamics. We combine the strengths of both techniques to measure the values and anisotropies of the electron and hole *g*-factors.

**Fig. 2:**
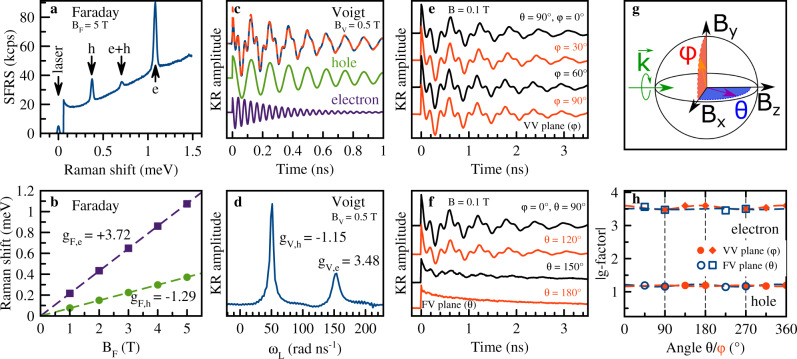
Pump-probe Kerr rotation and spin-flip Raman scattering of **FA**_**0.9**_**Cs**_**0.1**_**PbI**_**2.8**_**Br**_**0.2**_. **a** SFRS spectrum in Faraday geometry (*θ* = 0^∘^, *φ* = 0^∘^) measured at *T* = 1.6 K. The excitation/detection polarization is *σ*^+^/*σ*^−^. **b** Magnetic field dependencies of the measured Raman shifts (symbols). Lines are linear fits. **c** TRKR signal (blue line) measured in the Voigt geometry (*θ* = 90^∘^, *φ* = 0^∘^). Red dashed line is fit with two oscillatory functions (see Methods) with the parameters: *S*_e_/*S*_h_ = 0.8, ${T}_{{{{{{{{\rm{2,e}}}}}}}}}^{* }(B=0.5\,{{{{{{{\rm{T}}}}}}}})=275$ ps, ${T}_{{{{{{{{\rm{2,h}}}}}}}}}^{* }(B=0.5\,{{{{{{{\rm{T}}}}}}}})=690$ ps, *g*_*h*_ = − 1.15 and *g*_*e*_ = + 3.48. Decomposed electron and hole components are given below. TRKR data are measured at *T* = 5 K. **d** Fast Fourier transform spectrum of the signal from **c**. **e**, **f** TRKR signals in magnetic fields of 0.1 T rotated in the Voigt-Voigt and Faraday-Voigt planes, respectively. **g** Laboratory coordinate system: blue horizontal (red vertical) plane is the Faraday-Voigt (Voigt-Voigt) plane. **h** Angular dependences of the electron and hole *g*-factors for the magnetic field rotated in the Faraday-Voigt (open symbols) and Voigt-Voigt (closed symbols) planes. Note that while *g*_h_ < 0 we show ∣*g*_h_∣. Lines are guides to the eye. The indices V and F indicate the Voigt and Faraday directions of the magnetic field.

In presenting the experimental data, let us start with the FA_0.9_Cs_0.1_PbI_2.8_Br_0.2_ crystal. In the corresponding SFRS spectrum measured in the Faraday geometry at *B*_F_ = 5 T, pronounced lines associated with the electron and hole spin-flips are detected, see Fig. 2a. The larger shift of the electron line corresponds to a larger Zeeman splitting, i.e. to a larger absolute value of the electron *g*-factor compared to the hole *g*-factor. Analysis of the polarization properties in the Faraday and Voigt geometries allows us to conclude that the SFRS signals are provided by the spin-flip of a resident electron or hole, interacting with a photogenerated exciton^42^. The resident carriers are created by photogeneration, and are localized at separate crystal sites for cryogenic temperatures^28^. The specific feature of lead halide perovskites is the coexistence of resident electrons and holes, which is unusual for common semiconductors. Here, we make use of this feature, as in one crystal both the electron and hole properties can be studied in the same experiment. The e+h SFRS line in Fig. 2a has a shift corresponding to *g*_e_ + *g*_h_, evidencing that it is due to a combined spin-flip of an electron and a hole, interacting with the same exciton. From the linear dependence of the Raman shifts on magnetic field, using Eq. (2), we evaluate *g*_F,h_ = − 1.29 and *g*_F,e_ = + 3.72, Fig. 2b. Note that both shift dependencies show no offset at zero magnetic field, confirming the involvement of a resident carrier, as a finite shift would be expected due to electron-hole exchange interaction for carriers bound within an exciton^18^. Further we do not observe any deviations from the linear law [Eq. (2)] even at the smallest applied magnetic fields showing that the Rashba effect is negligible.

In pump-probe Kerr rotation (KR) experiments, carrier spin polarization along the light wave vector direction **k** is induced by the circularly polarized pump pulses, Fig. 2g and Methods. The spin polarization dynamics are detected via the Kerr rotation of the linearly-polarized probe pulses. Commonly, KR is measured in Voigt geometry where the Larmor spin precession about the magnetic field leads to an oscillating decaying signal. An example for *B*_V_ = 0.5 T is shown in Fig. 2c. The signal is contributed by two frequencies corresponding to *g*_h_ = − 1.15 and *g*_e_ = + 3.48, as can be seen from the Fast Fourier Transformation (FFT) spectrum in Fig. 2d and also from fitting it with two oscillating functions (Methods). The decomposed spin dynamics of holes and electrons are shown in Fig. 2c.

The *g*-factor anisotropy is measured by tilting the magnetic field in a vector magnet. The field direction is defined by two angles: *θ* for rotation in the Faraday-Voigt (FV) plane (blue) and *φ* for rotation in the Voigt-Voigt (VV) plane (red), see Fig. 2g. For VV plane rotation (**B**⊥**k**) the TRKR signals are very similar, see Fig. 2e. For rotation from Voigt to Faraday geometry (FV plane), see Fig. 2f, the spin precession amplitude decreases while the amplitude of the monotonically decaying signal increases. The latter corresponds to the signal in Faraday geometry (*θ* = 0^∘^ or 180^∘^) reflecting the longitudinal spin relaxation. The *g*-factors evaluated from Fig. 2e, f are collected in Fig. 2h. One can see that both the electron and hole *g*-factors are pretty isotropic in the FA_0.9_Cs_0.1_PbI_2.8_Br_0.2_ crystals, varying in the ranges from +3.48 to +3.60 and from −1.15 to −1.22, respectively.

**Fig. 3:**
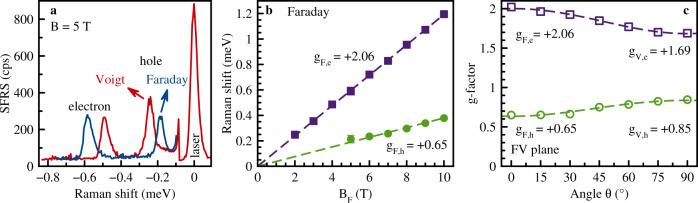
Spin-flip Raman scattering of CsPbBr_3_. **a** SFRS spectra measured in the Faraday (blue) and Voigt (red) geometries at *T* = 1.6 K. The excitation/detection polarization is *σ*^−^/*σ*^+^. Spectra are shown for the anti-Stokes spectral range where photoluminescence is suppressed. **b** Magnetic field dependence of the electron (purple squares) and hole (green circles) Stokes Raman shifts. Lines are linear fits with *g*_F,e_ = + 2.06 and *g*_F,h_ = + 0.65. **c** Angular dependences of the electron and hole *g*-factors for the magnetic field rotated in the Faraday-Voigt plane (*φ* = 0^∘^). Lines are fits using Eq. (4). The sample *c*-axis is oriented perpendicular to **k** and parallel to the x-axis, **c**∥**B**, corresponding to the Voigt geometry (*θ* = 90^∘^).

Let us turn to the CsPbBr_3_ crystal with the *c*-axis perpendicular to **k**. SFRS spectra measured in the Faraday and Voigt geometries are shown in Fig. 3a. They reveal a difference in the Raman shifts evidencing a pronounced anisotropy of the carrier *g*-factors: *g*_F,e_ = + 2.06, *g*_V,e_ = + 1.69 for the electrons and *g*_F,h_ = + 0.65, *g*_V,h_ = + 0.85 for the holes. The magnetic field dependencies of the Raman shifts presented in Fig. 3b together with the TRKR results for this sample published in ref. ^28^ allows us to conclude that in CsPbBr_3_, similarly to FA_0.9_Cs_0.1_PbI_2.8_Br_0.2_, the spin signals are contributed by resident carriers. The carrier spin dynamics of CsPbBr_3_ bulk single crystals is comparable to that of the other bulk single crystals discussed in this manuscript. For further details, we want to refer to ref. ^28^ containing an extended pump probe Kerr study of CsPbBr_3_ single crystals. The measured anisotropy in the FV plane is given in Fig. 3c. It can be well described by

4 $${g}_{{{{{{{{\rm{e(h)}}}}}}}}}(\theta ,\varphi ={0}^{\circ })=\sqrt{{g}_{{{{{{{{\rm{F,e(h)}}}}}}}}}^{2}{\cos }^{2}\theta +{g}_{{{{{{{{\rm{V,e(h)}}}}}}}}}^{2}{\sin }^{2}\theta }.$$

**Fig. 4:**
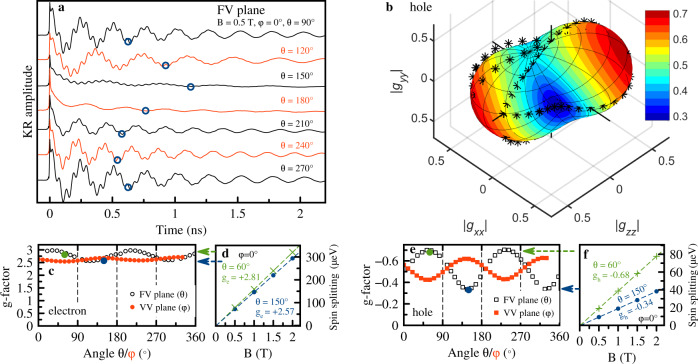
Pump-probe Kerr rotation in MAPbI_3_. **a** TRKR signals in magnetic fields of 0.5 T rotated in the Faraday-Voigt plane (*φ* = 0^∘^). *T* = 7 K. The variation of the hole Larmor frequency is indicated by the open circles. **b** Three-dimensional presentation of the hole *g*-factor tensor. Stars are experimentally measured values. The contour is a fit to the data using Eq. (4). **c**, **e** Angular dependencies of the electron and hole *g*-factors for the magnetic field rotated in the Faraday-Voigt (open symbols) and Voigt-Voigt (closed symbols) planes. **d**, **f** Magnetic field dependencies of the electron and hole Zeeman splittings evaluated from their Larmor frequencies. Experimental data (symbols) are measured at *θ* = 60^∘^ and 150^∘^ (*φ* = 0^∘^). Lines are linear fits.

In the MAPbI_3_ crystal, as well as in the two other materials, the spin precession of resident electrons and holes can be also well resolved in the TRKR signals, Fig. 4a. For rotation of the field orientation in the FV plane, a strong anisotropy of the hole *g*-factor can be concluded from the variation of the hole precession period, e.g., following the third minimum of the hole precession as indicated by the circles. The electron *g*-factor is also anisotropic. The *g*-factor variations for rotation in the FV and VV planes are given in Fig. 4c, e. For the holes it ranges between −0.28 and −0.71, while for the electrons is varies between +2.46 and +2.98. Interestingly, the *g*-factor extremal values do not coincide with the main cubic axes given by the angles *θ*, *φ* = {0^∘^, 90^∘^, 180^∘^, 270^∘^}. We have recorded a large data set by measurements for further angles not falling into the FV and VV planes and found that the main axis of the electron *g*-tensor has the orientation *θ* = 33^∘^, *φ* = 54^∘^ and for the hole *g*-tensor its direction is *θ* = 57^∘^, *φ* = 54^∘^. The hole *g*-tensor is visualized as three-dimensional plot in Fig. 4b, for the electron see the SI. SFRS measurements are also working well for the MAPbI_3_ crystal, yielding the same *g*-factor values as we show in SI.

## Discussion

**Fig. 5:**
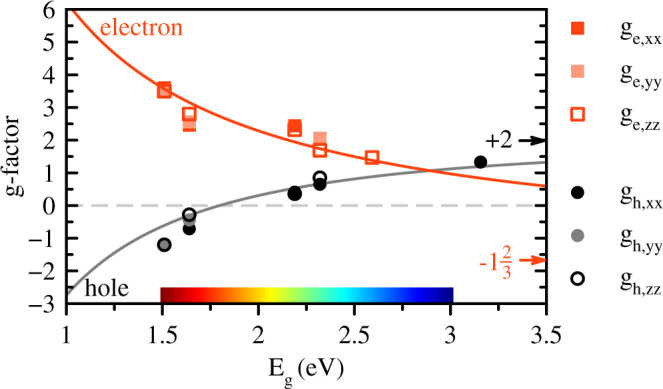
Electron and hole *g*-factors versus band gap energy in lead halide perovskite crystals. Experimental data are given by the symbols. Solid lines are fits with Eqs. (5) and (6) using the parameters Δ = 1.5 eV, ℏ*p*/*m*_0_ = 6.8 eVÅ, and Δ*g*_*e*_ = − 1. The limiting values of the electron (−5/3) and hole ( + 2) *g*-factors for *E*_*g*_ → *∞* are given by the arrows.

Our experimental results for the Landé factors of electrons and holes are summarized in the Table 1 and plotted in Fig. 5, where the *g*-factor tensor components are shown as function of the band gap. The clear correlation of the *g*-factors and the band gap energy requires a theoretical explanation. Note, that we do not observe any deviations from the linear Zeeman term [Eq. (2)] even in the smallest applied magnetic fields, hence we neglect the Rashba term in the analysis. To that end we use atomistic approaches based on the density functional theory (DFT) and empirical tight-binding method (ETB), to calculate the band structure and Landé factors of prototypical inorganic analogues of the studied perovskites: CsPbI_3_, CsPbBr_3_, and CsPbCl_3_^43^. Although these all-inorganic systems are different from organic-inorganic lead halides, resulting in somewhat different band gaps, spin-orbit couplings and Landé factors, the organic molecules are not crucial for the band structure formation, so that the study of the inorganic perovskites allows us to establish general trends and, eventually, formulate a semi-phenomenological model for the Zeeman splitting.

**Table 1:** Electron and hole *g* factors measured in the studied perovskite crystals at *T* = 1.6 and 5 K.

Material	*E*_*g*_ (eV)	*g*_e_	*P*_e_	*g*_h_	*P*_h_	Comments
FA_0.9_Cs_0.1_PbI_2.8_Br_0.2_	1.527	+3.48 to +3.60	2%	−1.15 to −1.22	<4%	isotropic
MAPbI_3_	1.652^53^	+2.46 to +2.98	10%	−0.28 to −0.71	43%	anisotropic, tilted
FAPbBr_3_	2.189	+2.32 to +2.44		+0.36 to +0.41		isotropic
CsPbBr_3_	2.352^28^	+1.69 to +2.06	10%	+0.65 to +0.85	13%	anisotropic
MAPb(Br_0.5_Cl_0.5_)_3_	2.592	+1.47				SFRS—z axis
MAPb(Br_0.05_Cl_0.95_)_3_	3.157			+1.33		TRKR—x axis

Minimum and maximum values are presented together with the degree of anisotropy defined as ${P}_{{{{{{{{\rm{e(h)}}}}}}}}}=100 \% \, \times ({g}_{\max }-{g}_{\min })/({g}_{\max }+{g}_{\min })$.

The details of the atomistic calculations and the ***k*** ⋅ ***p*** model description of the band structure are given in the SI. We express the components of the *g*-factor tensors via the matrix elements of the spin and orbital angular momenta of electrons and holes which can be further expressed via the interband momentum matrix elements and band gaps. The analysis of the influence from the different bands allows us to identify the key contributions to the electron and hole Landé factors. In particular, for the holes in the valence band the main contribution is related to the ***k*** ⋅ ***p*** mixing with the conduction band^18,19,38^ and, in the cubic phase one finds (see SI for details)

5 $${g}_{h}=2-\frac{4}{3}\frac{{p}^{2}}{{m}_{0}}\left(\frac{1}{{E}_{g}}-\frac{1}{{E}_{g}+{{\Delta }}}\right).$$

Here *p* is the interband matrix element of the momentum operator, *E*_*g*_ the band gap and Δ the spin-orbit splitting of the conduction band. For the electrons, the ***k*** ⋅ ***p*** mixing both with the top valence band and the remote valence states is important, resulting in (cf. ref. ^19^ and SI)

6 $${g}_{e}=-\frac{2}{3}+\frac{4}{3}\frac{{p}^{2}}{{m}_{0}{E}_{g}}+{{\Delta }}{g}_{e},$$

where Δ*g*_*e*_ is the remote band contribution. Eqs. (5) and (6) with the reasonably chosen parameters Δ = 1.5 eV, *ℏp*/*m*_0_ = 6.8 eV ⋅ Å, and Δ*g*_*e*_ = − 1 describe well the band gap dependence of the Landé factors across the studied perovskite classes, see Fig. 5. In agreement with experiment, the ***k*** ⋅ ***p***-mixing between the conduction and valence bands decreases with increasing band gap, thus the electron *g*-factor decreases with increasing *E*_*g*_ from large positive values down to − 2/3 + Δ*g*_*e*_ ≈ − 5/3, while the hole *g*-factor increases with increasing *E*_*g*_ from negative values up to +2. Note that in the studied range of band gap energies the separation from the remote bands is large, so that Δ*g*_*e*_ is practically independent of the material. Also the spin-orbit interaction is about constant since it is determined by the heavy lead atoms.

Let us now address the anisotropy of the *g*-factors observed mainly in the experiments on MAPbI_3_ and CsPbBr_3_. Overall, the *g*-factor anisotropy may be expected because at low temperatures the perovskites are known to undergo phase transitions from cubic to tetragonal or orthorhombic phases, see^34^. In the simplest approach the tetragonal and orthorhombic phases can be considered as a cubic phase distorted along the principal cubic axes *x*, *y*, and *z*. In the tetragonal phase *g*_*xx*_ = *g*_*yy*_ ≠ *g*_*zz*_, where *z* is the *C*_4_ axis. In the orthorhombic phase, on the other hand, *g*_*xx*_ ≠ *g*_*yy*_ ≠ *g*_*zz*_. The analysis shows (see SI) that the *g*-factor anisotropy is caused by the conduction band crystal splitting with Δ_*he*_ ≠ Δ_*le*_, see Fig. 1d, and by the anisotropy of the interband momentum matrix elements. The conduction band splitting affects mainly the hole *g*-factor [Eq. (5)], while the anisotropy of matrix elements affects both the electron and hole Landé factors, see the SI where details of the fits are presented. This, together with the fact that ∣*g*_*h*_∣ < *g*_*e*_, results in a larger anisotropy of the hole *g*-factors, *P*_*h*_ > *P*_*e*_, as observed in the experiment.

For the cubic, tetragonal and orthorhombic symmetries the main axes of the *g*-factor tensor are related to the cubic directions 〈001〉. We confirm this experimentally for CsPbBr_3_, FA_0.9_Cs_0.1_PbI_2.8_Br_0.2_ and FAPbBr_3_, while in MAPbI_3_ the main axes are found to be tilted with respect to the cubic axes. This might be a signature of the previously not reported transition to a monoclinic phase or a rotation of the crystallographic axes with respect to the lab frame^44–46^. In the former case, the reduced symmetry of the structure can be described by one of two possible point groups (both being subgroups of *D*_2*h*_): *C*_1_ (no non-trivial symmetry operations) or *C*_*i*_ (with space inversion) as symmetry operation. All other subgroups imply that at least one of the main axes of the *g*-factor tensor is the “cubic” axis [001], [010] or [100]. Alternatively, the explanation could be related to the formation or randomly oriented domains with different crystalline orientations. This question requires further experimental studies, particularly because X-ray diffraction for determination of the crystal structure at low temperatures is quite involved.

In conclusion, we have discovered experimentally and confirmed theoretically a universal dependence of the electron and hole *g*-factors on the band gap energy in the family of lead halide perovskite materials which is applicable for hybrid organic-inorganic and all-inorganic compounds. Our first-principles DFT calculations in combination with tight-binding and ***k*** ⋅ ***p*** approaches show that the universality originates from the electronic states that form the band gap being largely contributed by lead orbitals. Therefore, the *g*-factor dependence across the huge range of band gap energies from 1 eV up to 4 eV can be treated with the same band parameters. The derived parameters give insight into fundamental band properties, including band anisotropies caused by structural phase transitions. The selection of several halogen atoms (I, Br, Cl) and cation types (Cs, MA, FA) allows tailoring of the band gaps, which in turn leads to a correpsonding variation of the *g*-factor values. Our study provides a reliable relation to predict the Landé factors for the whole family of lead halide perovskites, including their nanostructures. This relation thus delivers the key parameter determining the spin physics of perovskites.

## Methods

### Samples

The class of lead halide perovskites possesses *A*Pb*X*_3_ composition, where the *A*-cation is typically Cs, methylammonium (MA, CH_3_NH_3_) or formamidinium (FA, CH(NH_2_)_2_) and the *X*-anion is a halogen Cl, Br, or I, giving rise of a huge flexibility. The latter is only limited by a favorable ratio of the anion to cation ion radii, named the Goldschmidt tolerance factor *t*, which should be close to unity^47^. By varying composition, the band gap of these perovskite materials can be tuned from the infrared up to the ultraviolet spectral range. All studied samples are lead halide perovskite single crystals grown out of solution with the inverse temperature crystallization (ITC) technique^9,48,49^. For the specific crystals the ITC protocols were modified.

### FA_0.9_Cs_0.1_PbI_2.8_Br_0.2_ crystals

*α*-phase FA_0.9_Cs_0.1_PbI_2.8_Br_0.2_ single crystals were grown in accordance with the method sketched above, for more details see ref. ^9^. First, a solution of CsI, FAI (FA being formamidinium), PbI_2_, and PbBr_2_, with GBL *γ*-butyrolactone as solvent is mixed. This solution is then filtered and slowly heated to 130 ^∘^C temperature, whereby the single crystals are formed in the black phase of FA_0.9_Cs_0.1_PbI_2.8_Br_0.2_. Afterwards the crystals are separated by filtering and drying. The *α*-phase (black phase) exhibits a cubic crystal structure at room temperature^50^. In the experiment the crystal was oriented with [001] pointing along the laser wave vector **k**. Note that the *g*-factor isotropy, the small shift of the PL line with temperature and further analyses^9,30^ suggest the typical lead halide perovskite crystal distortion from cubic symmetry to be small at small temperatures. The size of the crystal is ≈2 × 3 × 2 mm^3^. The crystal shape is non-cuboid, but the crystal structure exhibits aristotype cubic symmetry. Sample code: 515a.

### CsPbBr_3_ crystals

The CsPbBr_3_ crystals were grown with a slight modification of the ITC as stated above. Further information can be found in ref. ^48^. First, CsBr and PbBr_2_ were dissolved in dimethyl sulfoxide. Afterwards a cyclohexanol in N,N-dimethylformamide solution was added. The resulting mixture was heated in an oil bath to 105^∘^C whereby slow crystal growth appears. The obtained crystals were taken out of the solution and quickly loaded into a vessel with hot (100^∘^C) N,N-dimethylformamide. Once loaded, the vessel was slowly cooled down to about 50^∘^C. After that, the crystals were isolated, wiped with filter paper and dried. The obtained rectangular-shaped CsPbBr_3_ is crystallized in the orthorhombic modification. The crystals have one selected (long) direction along the *c*-axis [002] and two nearly identical directions along the $[\bar{1}10]$ and [110] axes^51^. The size of the crystal is ≈ 3 × 2 × 7 mm^3^. Sample code: DD4470/2.

### MAPbI_3_ crystals

Methylammonium (MA/CH_3_NH_3_) lead tri-iodine (MAPbI_3_) single crystals were low temperature solution-grown in a reactive inverse temperature crystallization (RITC) process, which utilizes a mixture of *γ*-butyrolactone GBL precursor solvent with alcohol^49^. The mixed precursor solvent polarity is changed compared to pure GBL, causing a lower solubility of MAPbI_3_ and an optimization of nucleation rates and centers, which result in an early crystallization at low temperatures. Black MAPbI_3_ single crystals were obtained at a temperature of 85^∘^C. At room temperature a tetragonal phase with lattice constants *a* = 0.893 nm and *c* = 1.25 nm was determined by XRD^49^. The size of the crystal is ≈4 × 3 × 2 mm^3^. The crystal shape is non-cuboid, but the crystal structure exhibits aristotype cubic symmetry. The front facet was X-ray characterized to point along the a-axis^49^. Sample code: MAPI-SC04.

### MAPb(Br_0.5_Cl_0.5_)_3_ & MAPb(Br_0.05_Cl_0.95_)_3_ crystals

In the case of MAPb(Br_0.5_Cl_0.5_)_3_, 0.3 mmol MACl, 0.3 mmol PbCl_2_, 0.7 mmol MABr and 0.7 mmol PbBr_2_ were dissolved in a mixture of 0.89 ml dimethylformamide (DMF) with 85 μl dimethyl sulfoxide (DMSO). In the case of MAPb(Br_0.05_Cl_0.95_)_3_, 2.2 mmol PbCl_2_, 1.2 mmol MACl and 1 mmol MABr were dissolved in 2 ml of 1:1 DMF:DMSO mixture. Both solutions were filtered through 0.45 μm polytetrafluoroethylene (PTFE) filter. The obtained solutions were slowly heated in an oil bath up to 62 ^∘^C. The crystals nucleate and grow in the temperature window 58–62 ^∘^C. The obtained crystals show a rectangular cuboid shape with sizes of 1.64 × 1.65 × 2.33 mm^3^ for MAPb(Br_0.05_Cl_0.95_)_3_ and 2 × 4 × 1 mm^3^ for MAPb(Br_0.5_Cl_0.5_)_3_. The MAPb(Br_0.05_Cl_0.95_)_3_ crystals are transparent and colorless, while the MAPb(Br_0.5_Cl_0.5_)_3_ crystals are transparent with a faint yellow appearance. Sample code: dd2924 and dd6347, respectively.

### FAPbBr_3_ crystals

The FAPbBr_3_ single crystals were grown with an analogous approach as the other samples following the ITC approach. Specific extended information are given in ref. ^52^. The crystal is of reddish transparent appearance and shows a rectangular cuboid shape with a size of 5 × 5 × 2 mm^3^. Sample code: OH0071a.

### Magneto-optical measurements

The samples were placed in a cryostat with the temperature variable from 1.6 K up to 300 K. For *T* = 1.6 K the sample is immersed in superfluid helium while for 4.2 K to 300 K the sample is in cooling helium gas. Two magnet cryostats equipped with split-coil superconducting solenoids were used. The first one is constructed to generate magnetic fields up to 10 T in a fixed direction. The second one, which is a vector magnet, has three pairs of orthogonal split coils, allowing us to apply magnetic fields up to 3 T in any direction. A sketch of the experimental geometry is shown in Fig. 2g. A magnetic field parallel to the light wave vector **k** is denoted here as **B**_*z*_ (Faraday geometry), magnetic fields perpendicular to **k** (Voigt geometry) are oriented in the plane spanned by the magnetic field axis *B*_*x*_ in the horizontal plane and *B*_*y*_ in the vertical direction. The angle *θ* is defined as the angle between **B**_*x*_ and **B**_*z*_ (for rotation in the Faraday-Voigt (FV) plane) with *θ*=0^∘^ corresponding to **B**_*z*_∥**k**. The angle *φ* defines the rotational orientation in the vertical plane (Voigt-Voigt (VV) plane) with *φ* = 0^∘^ for the horizontal x-axis and *φ* = 90^∘^ for the vertical y-axis.

### Pump-probe time-resolved Kerr rotation (TRKR)

The coherent spin dynamics were measured by a pump-probe setup, where pump and probe had the same photon energy, emitted from the same pulsed laser^40^. A titan-sapphire (Ti:Sa) laser emitted 1.5 ps long pulses with a spectral width of about 1 nm (1.5 meV) at a pulse repetition rate of 76 MHz (repetition period *T*_R_ = 13.2 ns). The laser photon energy was tunable in the spectral range of 1.265 − 1.771 eV (700 − 980 nm). It was set to the vicinity of the exciton resonance at the maximum of the Kerr rotation signal: at 1.513 eV for FA_0.9_Cs_0.1_PbI_2.8_Br_0.2_ and at 1.637 eV for MAPbI_3_. The laser beam was split into two beams (pump and probe). The probe pulses were delayed with respect to the pump pulses by a mechanical delay line. Both pump and probe beams were modulated using photo-elastic modulators (PEM). The probe beam was always linearly polarized and its amplitude was modulated at a frequency of 84 kHz. The pump beam helicity was modulated between *σ*^+^ and *σ*^−^ circular polarization at a frequency of 50 kHz. The polarization of the reflected probe beam was analyzed, via a lock-in technique, with respect to the rotation of its linear polarization (Kerr rotation). In finite transverse magnetic field, the Kerr rotation amplitude oscillates in time reflecting the Larmor spin precession of the carriers and decays at longer time delays. When both electrons and holes contribute to the Kerr rotation signal, which is the case for the studied perovskite crystals, the signal can be described as a superposition of two decaying oscillatory functions: ${A}_{{{{{{{{\rm{KR}}}}}}}}}={S}_{{{{{{{{\rm{e}}}}}}}}}\cos ({\omega }_{{{{{{{{\rm{L,e}}}}}}}}}t)\exp (-t/{T}_{{{{{{{{\rm{2,e}}}}}}}}}^{* })+{S}_{{{{{{{{\rm{h}}}}}}}}}\cos ({\omega }_{{{{{{{{\rm{L,h}}}}}}}}}t)\exp (-t/{T}_{{{{{{{{\rm{2,h}}}}}}}}}^{* })$. Here *S*_e(h)_ are the signal amplitudes that are proportional to the spin polarization of electrons (holes). The *g*-factors are evaluated from the Larmor precession frequency *ω*_L,e(h)_ by means of ∣*g*_e(h)_∣ = *ℏω*_L,e(h)_/(*μ*_B_*B*). It is also important to note, that TRKR provides information on the *g*-factor magnitude, but not on its sign. The same is true for the SFRS technique below. Information on the sign is obtained from model calculations, e.g. for the studied perovskites *g*_e_ > 0 is predicted, see SI. Also knowledge on the exciton *g*-factor, *g*_X_, and its sign can help to identify the hole *g*-factor sign using *g*_X_ = *g*_e_ + *g*_h_^28^. For standard TRKR measurements, the external magnetic field is applied in the Voigt geometry perpendicular to the light wave vector **k**. For measuring the *g*-factor anisotropy the magnetic field orientation is tuned to various angles *θ*, *φ* using the vector magnet, see Fig. 2g.

### Spin-flip Raman scattering (SFRS)

The SFRS technique allows one to measure directly the Zeeman splitting of the electron and hole spins from the spectral shift of the scattered light from the laser photon energy^39,42^. The energy shift is provided by the spin-flip of carriers, with the required energy taken from or provided by phonons. The typical shifts do not exceed 1 meV at the magnetic field of 10 T, which demands for the high spectral resolution provided by high-end spectrometers with excellent suppression of scattered laser light. The experiments were performed for samples in contact with pumped liquid helium at *T* = 1.6 K. We used resonant excitation in the vicinity of the exciton resonances in order to enhance the SFRS signal, obtained for the following laser photon energies: 1.500 eV for FA_0.9_Cs_0.1_PbI_2.8_Br_0.2_, 2.330 eV for CsPbBr_3_, and 1.635 eV for MAPbI_3_. The resonant Raman spectra were measured in the backscattering geometry with the incident laser excitation density between 1 and 5 Wcm^−2^. The scattered light was analyzed by a Jobin-Yvon U1000 double monochromator (1 meter focal length) equipped with a cooled GaAs photomultiplier and conventional photon counting electronics. The used spectral resolution of 0.2 cm^−1^ (0.024 meV) allowed us to measure the SFRS signals in close vicinity of the laser line for spectral shifts ranging from 0.1 to 3 meV. The spectra were measured in co-polarized (*σ*^+^/*σ*^+^) or cross-polarized (*σ*^+^/*σ*^−^ and *σ*^−^/*σ*^+^) circular polarizations of excitation and detection for the Faraday geometry (**B**∥**k**) and in crossed or parallel linear polarizations for the Voigt geometry (**B**⊥**k**), with the definition of the magnetic field axes and angles as stated above in Methods (Magneto-optical measurements). For data presentation we plot the Stokes-shifted lines (i.e. the lines shifted to lower energies from the laser) as positive shifts, while for the anti-Stokes lines negative values are taken. Note that in the anti-Stokes spectra possible contributions of photoluminescence are absent, as up-conversion at low temperatures is weak, so that spin-flip lines can be clearly identified. On the other hand, for the Stokes spectra larger Raman shifts in stronger magnetic fields can be observed.

## Supplementary information

Supplementary Information

## Data Availability

The data on which the plots within this paper are based and other findings of this study are available from the corresponding author upon justified request.

## Code availability

The code on which the calculations within this paper are based and other findings of this study are available from the corresponding author upon justified request.
