# Supplementary material for: The Landé factors of electrons and holes in lead halide perovskites: universal dependence on the band gap
Source: Nat Commun. 2022 Jun 2;13:3062. doi: 10.1038/s41467-022-30701-0 (PMC9163162; doi:10.1038/s41467-022-30701-0)
Supplement: Supplementary file 1 — Supplementary Information [file 41467_2022_30701_MOESM1_ESM.pdf]

Supplementary Information:

The Landé factors of electrons and holes in lead halide perovskites: universal dependence on the band gap

E. Kirstein,[1] D. R. Yakovlev,[1,2] M. M. Glazov,[2] E. A. Zhukov,[1,2] D. Kudlacik,[1] I. V. Kalitukha,[2] V. F. Sapega,[2] G. S. Dimitriev,[2] M. A. Semina,[2] M. O. Nestoklon,[2] E. L. Ivchenko,[2] D. N. Dirin,[3] O. Nazarenko,[3] M. V. Kovalenko,[3,4] A. Baumann,[5] J. Höcker,[5] V. Dyakonov,[5] and M. Bayer[1,2]

[1]*Experimentelle Physik 2, Technische Universität Dortmund, 44227 Dortmund, Germany*
[2]*Ioffe Institute, Russian Academy of Sciences, 194021 St. Petersburg, Russia*
[3]*Department of Chemistry and Applied Biosciences, Laboratory of Inorganic Chemistry, ETH Zürich, 8093 Zürich, Switzerland*
[4]*Department of Advanced Materials and Surfaces, Laboratory for Thin Films and Photovoltaics, Empa - Swiss Federal Laboratories for Materials Science and Technology, 8600 Dübendorf, Switzerland*
[5]*Experimental Physics VI, Julius-Maximilian University of Würzburg, 97074 Würzburg, Germany*

S1. THEORY

In this section we present details of our theoretical approach to calculate the band structure and Zeeman effect in perovskite crystals. We outline the microscopic DFT and tight-binding methods as well as the $\boldsymbol{k}\cdot\boldsymbol{p}$-model and formulate the atomistically-inspired procedure for the estimation of the g-factors in perovskites.

A. Preliminaries

According to the Bloch theorem the electron wavefunctions in a crystal can be recast in the form

$$\Psi_{n,s;\boldsymbol{k}}(\boldsymbol{r}) = \frac{e^{\mathrm{i}\boldsymbol{k}\boldsymbol{r}}}{\sqrt{\mathcal{V}}} u_{n,s;\boldsymbol{k}}(\boldsymbol{r}), \tag{S1}$$

where $\boldsymbol{r}$ is the real space coordinate, n enumerates the bands, s is the spin index, $\boldsymbol{k}$ is the quasi-wavevector, $\mathcal{V}$ is the normalization volume and $u_{n,s;\boldsymbol{k}}(\boldsymbol{r})$ is the periodic Bloch amplitude. In the presence of a weak magnetic field $\boldsymbol{B}$, such that all characteristic energies related to the field are much smaller than the energy separations between the bands, the Zeeman splitting of the electron states is given by the Hamiltonian with the matrix elements

$$\mathcal{H}_{Z,ss'} = \frac{1}{2} g_0 \mu_B (\boldsymbol{\sigma}_{ss'}\cdot\boldsymbol{B}) + \mu_B (\boldsymbol{L}_{ss'}\cdot\boldsymbol{B}). \tag{S2}$$

Here $s, s' = \pm 1/2$ are the spin indices, $g_0 = 2$ is the free-electron Landé factor, $\mu_B = |e|\hbar/(2m_0 c)$ is the Bohr magneton with m_0 being free-electron mass, e being the electron charge, and c being the speed of light, $\boldsymbol{\sigma}= (\sigma_x, \sigma_y, \sigma_z)$ is the vector composed of the Pauli matrices and $\boldsymbol{L} = -\mathrm{i}[\boldsymbol{r}\times(\partial/\partial\boldsymbol{r})]$ is the angular momentum operator. Combining the first and second terms, Eq. (S2) can be rewritten in the simple form

$$\mathcal{H}_Z = \frac{\mu_B}{2} g_{\alpha\beta}\sigma_\alpha B_\beta, \tag{S3}$$

where $\alpha, \beta = x, y, z$ denote the Cartesian components, $g_{\alpha\beta}$ are the elements of the g-factor tensor. Using the completeness relation for the Bloch functions one can recast the components of the g-factor tensor in the form, see e.g., Refs. [S1–S4]

$$g_{\alpha\beta}\sigma_{\alpha,ss'}B_\beta = \sum_{\gamma=x,y,z} B_\gamma \left(g_0 \sigma^\gamma_{ns,ns'} - \frac{2\mathrm{i}}{m_0} \sum_{\substack{m,t\\ \alpha,\beta}} \epsilon_{\alpha\beta\gamma} \frac{p^\alpha_{ns;mt} p^\beta_{mt;ns'}}{E_n - E_m} \right). \tag{S4}$$

Here summation over the repeated subscripts is implied, $\sigma^\gamma_{ns,ns'} = \langle n, s|\sigma_\gamma|ns'\rangle$, $\epsilon_{\alpha\beta\gamma}$ is the Levi-Civita symbol, E_n and E_m are the band energies at the corresponding point of the Brillouin zone, the $p^\alpha_{ns;mt}$ are the interband momentum operator matrix elements. In Eq. (S4) summation over all bands except for the selected band n is carried out, they

are enumerated by the orbital index m and the spin index t. For completeness we give the expression for the inverse effective mass tensor $m_{\alpha\beta}$ at a band extremum

$$\frac{1}{m_{\alpha\beta}} = \frac{\delta_{\alpha\beta}}{m_0} + \frac{1}{m_0^2}\sum_{m,t}\frac{p^{\alpha}_{ns;mt}p^{\beta}_{mt;ns} + p^{\beta}_{ns;mt}p^{\alpha}_{mt;ns}}{E_n - E_m}, \tag{S5}$$

where $\delta_{\alpha\beta}$ is the Kronecker symbol. Both the effective masses and Landé factors are determined by the set of the band structure parameters, namely, the band gaps and the interband momentum matrix elements. Thus, simultaneous calculation of both quantities by various methods allows one to improve the parametrization of the effective Hamiltonians [S5]. Note that if relativistic effects are included, the momentum matrix elements $p^{\alpha}_{ns;mt}$ should be replaced by the appropriate matrix elements of the operator $\boldsymbol{\pi}$ which includes the spin-orbit coupling term [S2].

B. Band structure in the tight-binding and DFT approaches

For a realistic description of the band structure of perovskites we use a tight-binding model based on DFT calculations, see Ref. [S6]. Such a combination of *ab initio* and *empirical* methods allows us to gain access to the details of the band structure and provides ground for a simplified $\boldsymbol{k}\cdot\boldsymbol{p}$-modeling of the bands. In our analysis, we consider the high-symmetry cubic structure. This is a good starting point: the energy bands of lower symmetry crystal phases may be then described as a folded and distorted band structure of this most symmetric phase [S7]. The standard approach for the description of the electronic properties of the cubic phase of the prototype hybrid organic-inorganic perovskite $CH_3NH_3PbI_3$ is to consider its all-inorganic analogue, $CsPbI_3$ [S7]. The DFT calculations are performed using the WIEN2k package [S8] with the modified Becke-Johnson exchange-correlation potential [S9] in Jishi parametrization [S10], for details see Ref. [S6]. Note that for cubic $CsPbI_3$ with the lattice constant 6.289 Å, this approach gives the band gap $E_g = 1.366$ eV, while the experimental value is $E_g = 1.65$ eV [S11]. The difference between the DFT results and experimental data for this material is attributed to the renormalization of the band structure by the electron-phonon interaction [S12] which is estimated as hundreds of meV [S13]. The DFT calculations of the band structure of cubic $CsPbBr_3$ and $CsPbCl_3$ are performed in the same way. The lattice constants, 5.992 Å and 5.605 Å, respectively, are taken from Ref. [S10]. The value of the radius of the muffin tin used is $R_{\rm MT} = 2.5$ Bohr, except for Cl in which case $R_{\rm MT} = 2.37$ Bohr. The parameters controlling the numerical precision are $R_{\rm MT}\cdot K_{\rm MAX} = 13$ and $E_{\rm MAX} = 14$. With these parameters, the band gap for the cubic phase is $E_g = 2.52$ eV for $CsPbBr_3$ and $E_g = 3.09$ eV for $CsPbCl_3$. The dispersions for the $CsPbBr_3$ cubic crystal calculated by the DFT approach are shown by the green dashed curves in Fig. S1.

For the tight-binding calculations, we use the empirical tight-binding model with the $sp^3d^5s^*$ basis in the nearest neighbor approximation. It gives a precise description of the band structure of bulk III–V [S14] and group IV [S15] semiconductors. Recently, it has been shown that the extended $sp^3d^5s^*$ tight-binding method can be used to describe the band structure of inorganic perovskites with a meV-range precision [S6]. For $CsPbI_3$ we refer to the parameters from Ref. [S6]. For the two other perovskites, $CsPbBr_3$ and $CsPbCl_3$, we use the results of the tight-binding fit to the DFT approach outlined in Ref. [S6], they are given in Table S1; these materials are not stable in the cubic phase so that corresponding experimental data are not available. Note that the relative error in the DFT band gap is significantly smaller for Br- and Cl-based materials since their band gap is much larger. The corresponding tight-binding dispersion curves for $CsPbBr_3$ are shown in Fig. S1 by the solid thin black lines and demonstrate good agreement with the DFT calculations.

C. Effective Hamiltonian model

In this section we present the basic information about the $\boldsymbol{k}\cdot\boldsymbol{p}$ model applied to calculation of the conduction and valence band states in bulk perovskites and their key properties: the effective masses and g-factors. Following Refs. [S16–S18] we start our description with the minimum model which includes the topmost two-fold degenerate valence band and the nearest conduction bands. In the high-temperature cubic crystalline modification of the perovskites the direct band gap is formed at the R-point of the Brillouin zone (corner of the cube in the [111] direction). The schematics of the band structure in the vicinity of the R-point are shown in Fig. S2(a). For a tetragonal perovskite the bands are folded and the band gap is located at the Γ point of the Brillouin zone due to the band folding [S19], Fig. S2(b).

Table S1: Tight-binding parameters fitted to the DFT calculations. All values are given in eV. In addition to the parameters presented in the table, the parameters $s_c s_a^* \sigma$, $s_a^* d_c \sigma$, $p_c d_a \sigma$, $p_a d_c \pi$ and those involving the s_a and s_c^* orbitals are taken to be zero. The parameters of $CsPbI_3$ are reproduced from Ref. [S6].

	$CsPbI_3$	$CsPbBr_3$	$CsPbCl_3$
E_{sc}	−5.7767	−4.9645	−4.5816
E_{s^*a}	19.6780	19.7944	8.6310
E_{pa}	−2.3350	−2.5653	−3.4496
E_{pc}	4.4825	5.5804	6.4591
E_{da}	10.8491	12.9468	14.1585
E_{dc}	13.9357	15.9568	15.9638
$s_c p_a \sigma$	1.0421	1.0288	1.1429
$s_a^* p_c \sigma$	2.7092	2.5482	1.3349
$s_c d_a \sigma$	0.3749	−0.7094	0.7972
$pp\sigma$	−1.8838	−1.8488	−2.0710
$pp\pi$	0.1955	0.1990	0.1183
$p_a d_c \sigma$	1.0341	−1.1460	1.4326
$p_c d_a \pi$	−0.7960	−0.7768	−0.9618
$dd\sigma$	−1.1231	−1.1429	−1.1768
$dd\pi$	2.0000	2.0000	2.0000
$dd\delta$	−1.4000	−1.4000	−1.4000
$\Delta_a/3$	0.3250	0.0944	0.2290
$\Delta_c/3$	0.4892	0.5469	0.7071

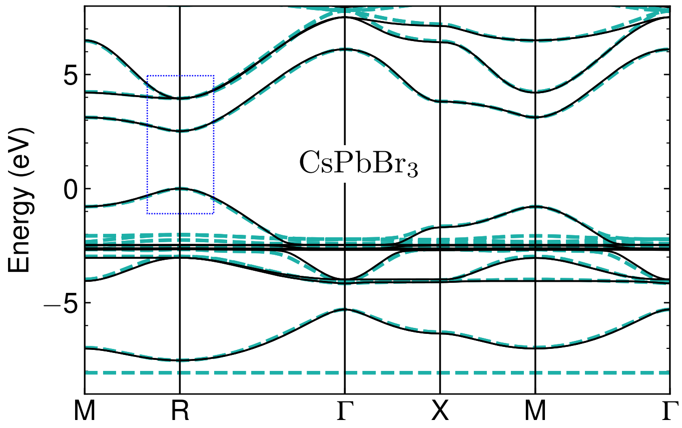

Figure S1: Comparison of the $CsPbBr_3$ band structure calculated in DFT and in the empirical tight-binding method. The DFT calculations are shown by the green dashed lines, the tight-binding results are given by the thin black lines. The blue dotted rectangle shows the topmost valence band and the lowest conduction bands in the vicinity of the R-point, see Fig. S2(a).

In both the cubic and tetragonal cases the valence band Bloch amplitudes can be recast in the form

$$\text{valence band:} \quad \begin{cases} u_{v,\frac{1}{2}}(\boldsymbol{r}) = \mathrm{i}\mathcal{S}(\boldsymbol{r})\uparrow, \\ u_{v,-\frac{1}{2}}(\boldsymbol{r}) = \mathrm{i}\mathcal{S}(\boldsymbol{r})\downarrow, \end{cases} \tag{S6a}$$

where $\uparrow, \downarrow$ denote the basic spinors, and $\mathcal{S}(\boldsymbol{r})$ is the invariant function. The conduction band states are formed from the three orbital Bloch amplitudes $\mathcal{X}(\boldsymbol{r})$, $\mathcal{Y}(\boldsymbol{r})$, and $\mathcal{Z}(\boldsymbol{r})$ which transform as the corresponding coordinates: in the cubic modification the axes $x \parallel [100]$, $y \parallel [010]$, $z \parallel [001]$ are equivalent, and in the tetragonal perovskite the z-axis is the C_4-axis with x and y being equivalent. The Bloch states are determined by the interplay of the crystalline

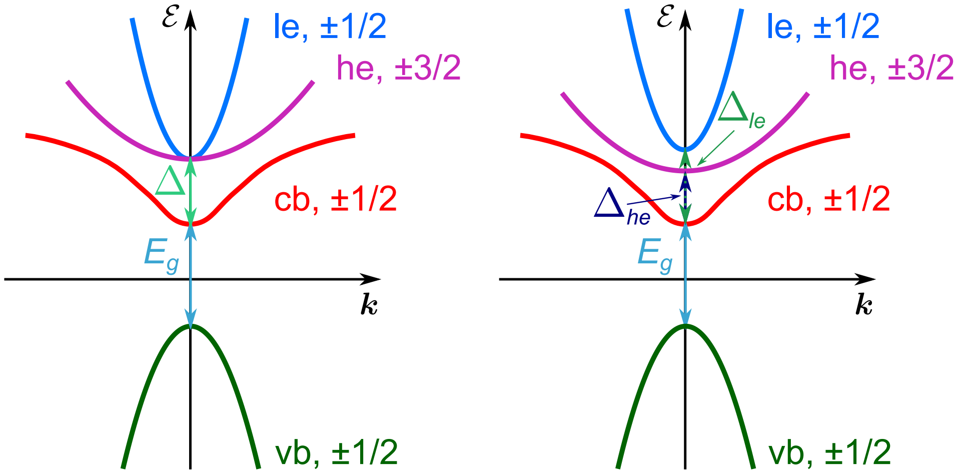

Figure S2: Schematic illustration of the band structure of a bulk (a) cubic perovskite crystal in the vicinity of the R-point of the Brillouin zone and (b) tetragonal perovskite crystal in the vicinity of the Γ-point.

splitting and spin-orbit interaction and can be described as

$$\text{bottom conduction band (c.b.):}\quad \begin{cases} u_{c,\frac{1}{2}}(\boldsymbol{r}) = -\sin\vartheta\,\mathcal{Z}(\boldsymbol{r})\!\uparrow - \cos\vartheta\,\dfrac{\mathcal{X}(\boldsymbol{r})+\mathrm{i}\,\mathcal{Y}(\boldsymbol{r})}{\sqrt{2}}\!\downarrow,\\ u_{c,-\frac{1}{2}}(\boldsymbol{r}) = \sin\vartheta\,\mathcal{Z}(\boldsymbol{r})\!\downarrow - \cos\vartheta\,\dfrac{\mathcal{X}(\boldsymbol{r})-\mathrm{i}\,\mathcal{Y}(\boldsymbol{r})}{\sqrt{2}}\!\uparrow, \end{cases} \tag{S6b}$$

$$\text{excited (light electron) c.b.:}\quad \begin{cases} u_{le,\frac{1}{2}}(\boldsymbol{r}) = \cos\vartheta\,\mathcal{Z}(\boldsymbol{r})\!\uparrow - \sin\vartheta\,\dfrac{\mathcal{X}(\boldsymbol{r})+\mathrm{i}\,\mathcal{Y}(\boldsymbol{r})}{\sqrt{2}}\!\downarrow,\\ u_{le,-\frac{1}{2}}(\boldsymbol{r}) = \cos\vartheta\,\mathcal{Z}(\boldsymbol{r})\!\downarrow + \sin\vartheta\,\dfrac{\mathcal{X}(\boldsymbol{r})-\mathrm{i}\,\mathcal{Y}(\boldsymbol{r})}{\sqrt{2}}\!\uparrow, \end{cases} \tag{S6c}$$

$$\text{excited (heavy electron) c.b.:}\quad \begin{cases} u_{he,\frac{3}{2}}(\boldsymbol{r}) = -\dfrac{\mathcal{X}(\boldsymbol{r})+\mathrm{i}\,\mathcal{Y}(\boldsymbol{r})}{\sqrt{2}}\!\uparrow,\\ u_{he,-\frac{3}{2}}(\boldsymbol{r}) = \dfrac{\mathcal{X}(\boldsymbol{r})-\mathrm{i}\,\mathcal{Y}(\boldsymbol{r})}{\sqrt{2}}\!\downarrow, \end{cases} \tag{S6d}$$

In the cubic modification the light and heavy electron states at the R-point are degenerate, Fig. S2(a). In the tetragonal modification a splitting between the light and heavy electrons arises, as Fig. S2(b). We introduce the band gap E_g as the energy gap between the bottom conduction band and the valence band, $E_g+\Delta_{le}$ is the gap between the light electron band and valence band, $E_g+\Delta_{he}$ is the gap between the heavy electron band and the valence band, see Fig. S2(b). The interband momentum matrix elements defined by (we assume that $p_\parallel$ and $p_\perp$ are real) are given by

$$p_\perp = \mathrm{i}\langle\mathcal{X}\,|p_x|\,\mathcal{S}\rangle = \mathrm{i}\langle\mathcal{Y}\,|p_y|\,\mathcal{S}\rangle,\quad p_\parallel = \mathrm{i}\langle\mathcal{Z}\,|p_z|\,\mathcal{S}\rangle. \tag{S7}$$

The parameter ϑ determines the relation between the crystalline splitting and the spin-orbit interaction. Naturally, in the cubic approximation $\cos\vartheta = \sqrt{2/3}$, $\sin\vartheta = 1/\sqrt{3}$, $\Delta_{le} = \Delta_{he} \equiv \Delta$, and $p_\parallel = p_\perp \equiv p$. In the quasi-cubic approximation [S2, S17, S18] the anisotropy parameters can be expressed in terms of the bare spin-orbit splitting energy Δ_{so} and the crystal field splitting Δ_c as

$$\Delta_{le} = \sqrt{\Delta_{so}^2 + \Delta_c^2 - \frac{2}{3}\Delta_{so}\Delta_c},\quad \Delta_{he} = \frac{\Delta_{le}+\Delta_{so}+\Delta_c}{2},\quad \tan 2\vartheta = \frac{2\sqrt{2}\Delta_{so}}{\Delta_{so}-3\Delta_c}. \tag{S8}$$

Within this minimum model we calculate the effective masses and g-factors for electrons and holes. For the electron at the bottom of the conduction band $(c, \pm 1/2)$, see Fig. S2, we obtain:

$$\frac{1}{m_{e\parallel}} = \frac{1}{m_0} + \frac{2p_\parallel^2}{m_0^2}\frac{\sin^2\vartheta}{E_g}, \tag{S9a}$$

$$\frac{1}{m_{e\perp}} = \frac{1}{m_0} + \frac{p_\perp^2}{m_0^2}\frac{\cos^2\vartheta}{E_g}. \tag{S9b}$$

Here the symbols $\parallel$ and $\perp$ denote the direction of the electron propagation, namely, along and perpendicular to the $C_4 \parallel z$ axis, respectively. In the valence band the hole effective masses take the form

$$-\frac{1}{m_{h\parallel}} = \frac{1}{m_{v.b.\parallel}} = \frac{1}{m_0} - \frac{2p_\parallel^2}{m_0^2}\left(\frac{\sin^2\vartheta}{E_g} + \frac{\cos^2\vartheta}{E_g+\Delta_{le}}\right), \tag{S10a}$$

$$-\frac{1}{m_{h\perp}} = \frac{1}{m_{v.b.\perp}} = \frac{1}{m_0} - \frac{p_\perp^2}{m_0^2}\left(\frac{\cos^2\vartheta}{E_g} + \frac{\sin^2\vartheta}{E_g+\Delta_{le}} + \frac{1}{E_g+\Delta_{he}}\right). \tag{S10b}$$

Note that the hole masses, m_h, have opposite signs as compared to the valence band electron masses, $m_{v.b.}$. Evidently, for the cubic crystal the effective masses and Landé factors become isotropic. In particular,

$$\frac{1}{m_{e\parallel}} = \frac{1}{m_{e\perp}} = \frac{1}{m_0} + \frac{2p^2}{m_0^2 E_g}, \quad -\frac{1}{m_{h\parallel}} = -\frac{1}{m_{h\perp}} = \frac{1}{m_0} - \frac{2p^2}{3m_0^2}\frac{3E_g+\Delta}{E_g(E_g+\Delta)}. \tag{S11}$$

Equations (S10) transform into the standard expression (2.48) of Ref. [S3] for cubic III-V semiconductors when neglecting the non-parabolicity and making the natural replacements $E_g \to -(E_g+\Delta)$, $E_g+\Delta \to -E_g$ that are related to the difference in the band order in perovskites and GaAs-like crystals.

We now turn to the Landé factors. For the bottom conduction band one has

$$g_{e\parallel} = -\frac{2}{3} + \frac{2p_\perp^2}{m_0}\frac{\cos^2\vartheta}{E_g}, \tag{S12a}$$

$$g_{e\perp} = -\frac{2}{3} + \frac{2\sqrt{2}p_\parallel p_\perp}{m_0}\frac{\cos\vartheta\sin\vartheta}{E_g}. \tag{S12b}$$

Here the subscripts $\parallel$ and $\perp$ of the Landé factors denote the direction of the magnetic field with respect to the C_4 axis. In these expressions we took into account the difference from the value of 2 of the free-electron spin contribution to the g-factor due to the mixed form of wavefunctions (S6b), see the first term on the r.h.s. of Eq. (S4). For the valence band we obtain

$$g_{h\parallel} = 2 - \frac{2p_\perp^2}{m_0}\left(\frac{\cos^2\vartheta}{E_g} + \frac{\sin^2\vartheta}{E_g+\Delta_{le}} - \frac{1}{E_g+\Delta_{he}}\right), \tag{S13a}$$

$$g_{h\perp} = 2 - \frac{2\sqrt{2}p_\parallel p_\perp}{m_0}\cos\vartheta\sin\vartheta\left(\frac{1}{E_g} - \frac{1}{E_g+\Delta_{le}}\right). \tag{S13b}$$

Note that the valence band g-factors in the electron and hole representation have the same sign because the transformation from the electron to the hole representation includes both a change in the sign of energy and the time reversal. We define the Landé factor in such a way that, e.g., for $\boldsymbol{B} \parallel z$ the splitting $E_{+1/2} - E_{-1/2}$ between the states with spin projection $+1/2$ and $-1/2$ onto the z axis is given by $g_{e\parallel}\mu_B B_z, g_{h\parallel}\mu_B B_z$, see Eq. (S3).

The expressions for the valence band g-factor in the cubic limit transform into the well-known formula (2.48) of Ref. [S3] with the same replacements $E_g \to -(E_g+\Delta)$, $E_g+\Delta \to -E_g$ as for the effective mass. The contributions due to the $\boldsymbol{k}\cdot\boldsymbol{p}$ interaction of the conduction and valence bands are also in agreement with Yu [S16], both in terms of magnitudes and signs. When comparing with Ref. [S16], one has to keep in mind that in the notations of Yu the energy is reckoned from the heavy electron band and $P_{\parallel,\perp} = (\hbar/m_0)p_{\parallel,\perp}$. Also $\hbar^{-2}$ is omitted in Eqs. (11)-(14) of Ref. [S16].

Importantly, in Ref. [S16] the remote band contributions to the conduction band Landé factors were included through the 'magnetic' Luttinger parameters $\kappa_{1,2}$. The importance of the remote band contributions for the conduction band parameters is highlighted by our microscopic calculations, see below. To illustrate their role let us estimate, within the cubic approximation, the contribution of the remote band with the orbital Bloch functions $\mathcal{XY}$, $\mathcal{XZ}$, and $\mathcal{YZ}$

(F_1^+ or R_5^+) to the effective mass and g-factor of the conduction band electron. The importance of this band will be clarified below from the comparison of the $\boldsymbol{k}\cdot\boldsymbol{p}$-method with the atomistic approaches. Due to their even parity, these bands do not contribute to the hole mass and Landé factor. We denote

$$q = \langle \mathcal{XY}|p_x|\mathcal{Y}\rangle = \langle \mathcal{XY}|p_y|\mathcal{X}\rangle, \text{ etc.}, \tag{S14}$$

and select the phases of the wavefunctions in such a way that q is real. We also neglect the spin-orbit splitting of the remote band as compared to the distance E_g' between the remote band and the conduction band. The contribution to the inverse effective mass reads

$$\Delta\left(m_e^{-1}\right) = \frac{4}{3}\frac{q^2}{m_0^2 E_g'} \quad \Rightarrow \quad \frac{1}{m_e} = \frac{1}{m_0} + \frac{2p^2}{m_0^2}\frac{1}{3E_g} + \frac{4}{3}\frac{q^2}{m_0^2 E_g'}, \tag{S15}$$

while the contribution to the Landé factor takes the form

$$\Delta g_e = -\frac{4}{3}\frac{q^2}{m_0 E_g'}. \tag{S16}$$

In the cubic approximation this correction is isotropic, the difference of the remote bands contribution to the Landé factor $\Delta g_{e\parallel} - \Delta g_{e\perp}$ appears due to the crystalline splitting of the remote F_1^+ orbitals and the anisotropy of the matrix elements in Eq. (S14).

D. Results

To compute the g-factor in the empirical tight-binding (ETB) model we directly use Eq. (S4). We construct the tight-binding Hamiltonian in the R-point of the Brillouin zone, and find the eigenvalues as well as the eigenvectors that represent the energies and wave functions at the R-point. The resulting eigenvectors should be "symmetrized" to form the canonical basis of irreducible representations in the R point (R_6^+ for the valence band and R_6^- for the conduction band [S7]), in order to exclude an arbitrary phase of the computed eigenvectors from the g-factor values. Then, in this basis, we calculate the matrix elements of the spin operator and the velocity operator in the standard manner [S20], and use them in Eq. (S4) to find the g-factors.

Table S2: Band gap, spin-orbit splitting, effective masses and g-factors for different perovskite materials extracted from DFT and ETB calculations. The band gap and spin-orbit splitting from DFT are reproduced in the ETB exactly, while there is a small difference in the masses. The g-factors are extracted only from the ETB calculations. Subscripts e and h denote the bottom conduction band electron states and the top valence band hole states.

	E_g (eV)	Δ (eV)	m_h/m_0 (DFT)	m_h/m_0 (ETB)	m_e/m_0 (DFT)	m_e/m_0 (ETB)	g_h (ETB)	g_e (ETB)
$CsPbI_3$	1.366	1.266	0.16	0.191	0.18	0.184	−0.108	1.660
$CsPbBr_3$	2.520	1.431	0.26	0.298	0.30	0.291	1.343	0.127
$CsPbCl_3$	3.090	1.526	0.28	0.348	0.36	0.398	1.527	−0.080

The calculated values of the Landé factors are summarized in Tab. S2. It also presents the values of the effective masses found by fitting the numerically calculated dispersion curves with parabolas in the vicinity of the R-point. Note that the direct application of Eq. (S5) yields similar values for the conduction band effective masses, but larger values for the valence band effective masses because of the k^2 diagonal terms appearing in the tight-binding Hamiltonian, see the discussion in Ref. [S20]. We note that the g-factor dependence on the band gap corresponds well to the experiment. The incomplete agreement with the measured data of the g-factors are mainly related to the difference in the band gap energies of our DFT and ETB atomistic calculations and those observed for the studied crystals, as well as to limitations in the extraction of the momentum matrix elements in the state-of-the-art DFT→ ETB procedures. The open circles in Fig. S3 show the Landé factors from the ETB procedure as function of the band gap energy. It is seen that the atomistic approach gives reasonable values of the Landé factor with the right trends: the electron g-factor decreases with increasing band gap energy E_g, while the hole g-factor increases with increasing E_g.

To gain further insight into the key band parameters and to provide atomistic empirical expressions for the g-factors within the effective Hamiltonian model we have analyzed the contributions of the different bands to the effective masses and g-factors, Fig. S4. Namely, we evaluated the different terms in Eqs. (S4) and (S5) resulting from band mixing and plotted them in arbitrary units as function of the energy E_m of the corresponding state

involved in the summation. Larger values indicate larger contributions to the inverse effective mass and Landé factor. The calculations clearly show that, for the valence band, the effective mass and the g-factor are dominated by the contribution of the conduction band (blue rectangle in Fig. S4, left panels). For the bottom conduction band, both the topmost valence band (magenta) and the remote valence bands (yellow) are important. Furthermore, it follows from our parametrization that the Kane matrix element $P = (\hbar/m_0)p$ depends weakly on the material ranging from 4.4 eV·Å to 5.5 eV·Å. This enables us to use the atomically-inspired effective $\boldsymbol{k}\cdot\boldsymbol{p}$-approach formulated in Sec. S1 C to evaluate the Landé factors for the perovskite crystals in the cubic phase. To that end we use Eq. (S13)

$$g_h = 2 - \frac{4p^2}{3m_0}\left(\frac{1}{E_g} - \frac{1}{E_g+\Delta}\right), \tag{S17}$$

for the hole g-factor and a combination of Eqs. (S12) and (S16)

$$g_e = -\frac{2}{3} + \frac{4p^2}{3m_0E_g} + \Delta g_e, \tag{S18}$$

for the electron g-factor. We recall that $p = p_\parallel = p_\perp$ in the cubic case. Taking $\Delta = 1.5$ eV, $P = \hbar p/m_0 = 5.1$ eV·Å, and $\Delta g_e = -0.94$ we simultaneously reproduce the band gap dependence of the Landé factors found in the ETB approach, see the dashed lines in Fig. S3. Equations (S17) and (S18) clearly highlight the physics behind the dependences: The contributions to the g-factors due to the conduction band-valence band mixing have opposite signs for the electron (positive) and hole (negative) and decrease in absolute value with increasing band gap energy. That is why the electron g-factor decreases with increasing E_g (from large positive values at small E_g to $-2/3 + \Delta g_e$) and the hole g-factor increases (from large negative values, it passes through zero and reaches +2 at large band gaps).

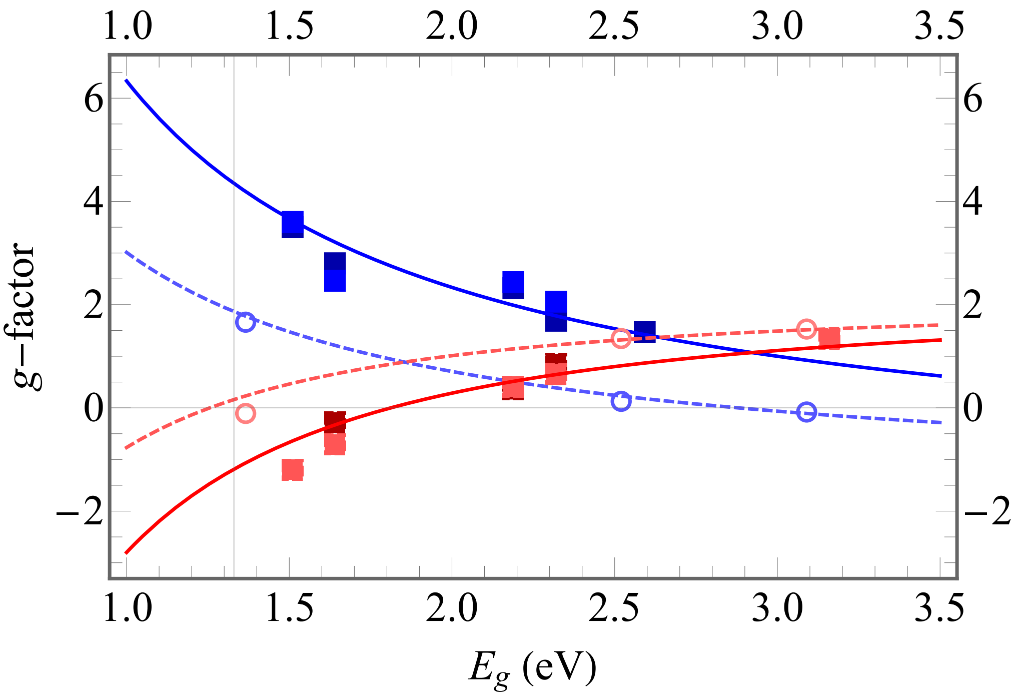

Figure S3: Summary of the calculated and measured Landé factors for electrons and holes in the perovskites as function of the band gap. Blue symbols and lines show the electron g-factors, red symbols and lines show the hole g-factors. Open circles are ETB calculations from Table S2. Dashed lines are calculated within the atomically-inspired $\boldsymbol{k}\cdot\boldsymbol{p}$-approach after Eqs. (S17) and (S18) with $\Delta = 1.5$ eV, $P = \hbar p/m_0 = 5.1$ eVÅ, and $\Delta g_e = -0.94$, closely matching the ETB calculations. Filled squares show the experimental data (see main text for details). Solid lines are calculated within the $\boldsymbol{k}\cdot\boldsymbol{p}$-approach after Eqs. (S17) and (S18) with $\Delta = 1.5$ eV, $P = \hbar p/m_0 = 6.8$ eVÅ, and $\Delta g_e = -1$ in reasonable agreement with the experiment.

Choosing reasonable values $P = \hbar p/m_0 = 6.8$ eV·Å, $\Delta = 1.5$ eV, and $\Delta g_e = -1$, we obtain good agreement with the experimental data, see the solid lines in Fig. S3.

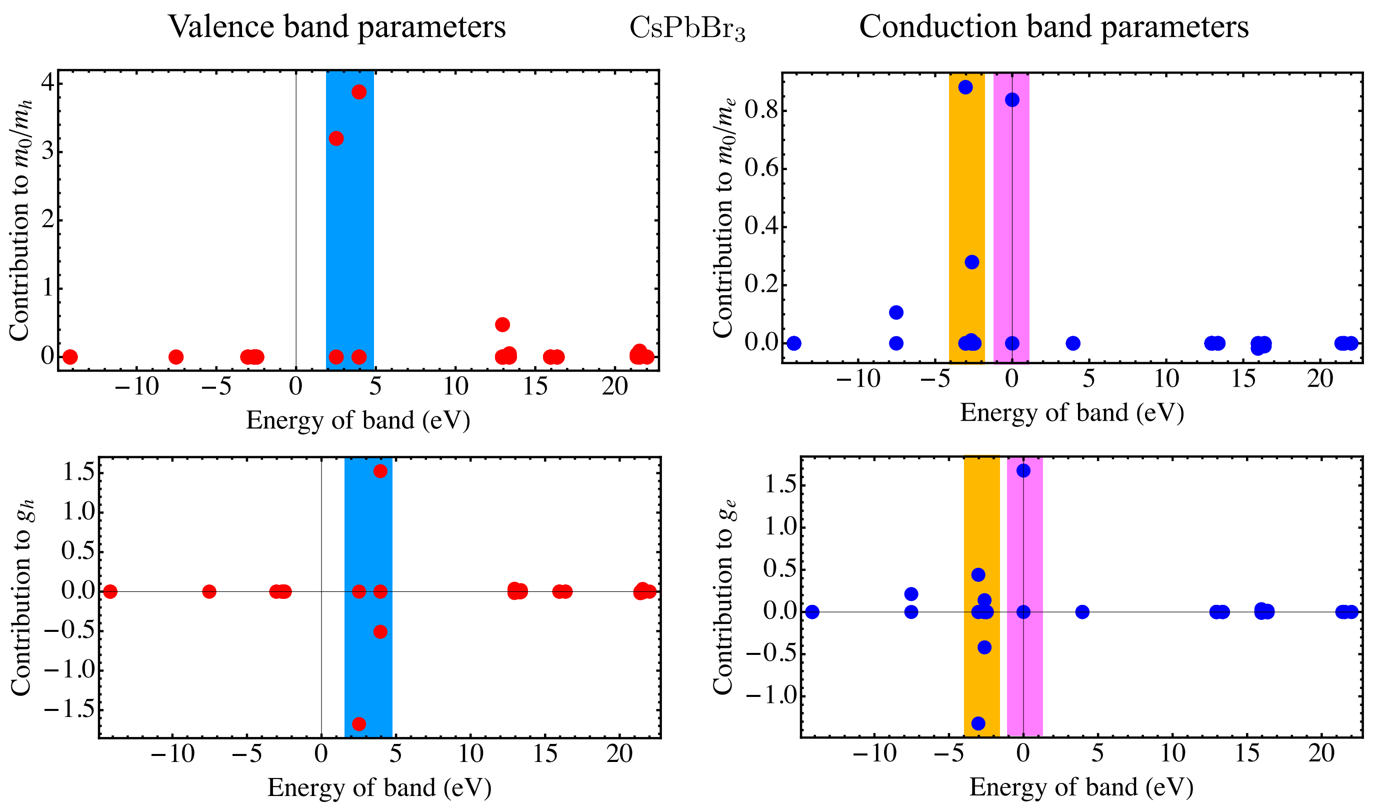

Figure S4: Contributions to the effective masses and g-factors from various bands calculated for $CsPbBr_3$ after Eqs. (S4) and (S5). Blue, magenta and yellow rectangles show the energy ranges of the conduction band, the topmost valence band and a bunch of remote valence bands of F_1^+ symmetry, respectively. The contributions are given in arbitrary units.

Using Eqs. (S12) and (S13) we evaluate also the bright exciton g-factor which describes the splitting of the exciton radiative doublet into circularly polarized components, by $g_X = g_e + g_h$. For the magnetic field along the main axis we have

$$g_{X\parallel} = \frac{4}{3} - \frac{2p_\perp^2}{m_0}\left(\frac{\sin^2\vartheta}{E_g + \Delta_{le}} - \frac{1}{E_g + \Delta_{he}}\right) + \Delta g_e, \tag{S19a}$$

$$g_{X\perp} = \frac{4}{3} + \frac{2\sqrt{2}p_\parallel p_\perp}{m_0}\frac{\cos\vartheta\sin\vartheta}{E_g + \Delta_{le}} + \Delta g_e. \tag{S19b}$$

It is noteworthy that the contributions to the individual g-factors due to the $\boldsymbol{k}\cdot\boldsymbol{p}$-mixing of the valence band with the bottom conduction band $\propto 1/E_g$ cancel in the exciton g-factor.

E. g-factor anisotropy in $CsPbBr_3$

It is instructive to analyze in more detail the anisotropy of the g-factor components for the case of $CsPbBr_3$, where this anisotropy is most clearly pronounced. in particular, the experiment shows that

$$g_{e\parallel} = 1.69, \quad g_{e\perp} = 2.06; \quad g_{h\parallel} = 0.85, \quad g_{h\perp} = 0.65. \tag{S20}$$

Our model has the following parameters: $p_\parallel$, $p_\perp$, Δ_{le}, Δ_{he}, and ϑ. The interband momentum matrix elements $p_\parallel$ and $p_\perp$ can be considered as independent parameters, but the three remaining parameters which deteremine the conduction band structure, can be expressed by virtue of Eq. (S8) via two energies, namely the spin-orbit energy Δ_{so} and the crystalline splitting Δ_c. The four parameters $(p_\parallel, p_\perp, \Delta_{so}, \Delta_c)$ can be determined via the four measured values of the g-factors, Eq. (S20), and the band gap energy $E_g = 2.352$ eV.

We use a least squares fit and find the parameter set to be

$$P_\perp = \frac{\hbar p_\perp}{m_0} = 6.55 \text{ eV} \cdot \text{Å}, \quad P_\parallel = \frac{\hbar p_\parallel}{m_0} = 7.92 \text{ eV} \cdot \text{Å}, \quad \Delta_{so} = 1.29 \text{ eV}, \quad \Delta_c = -0.11 \text{ eV}, \tag{S21}$$

which yields $\cos^2\vartheta \approx 0.7$, $\Delta_{le} = 1.34$ eV, and $\Delta_{he} = 1.26$ eV. The obtained values of the g-factors coincide with the experimental data, see Eq. (S20), within the numerical accuracy.

In this work, we abstain from a precise fitting of the parameters for all perovskites, since, for their unambiguous determination, DFT+ETB calculations are needed for low-symmetry phases on the theory side, and a detailed analysis of the g-factor anisotropy across different perovskites on the experimental side.

S2. ADDITIONAL EXPERIMENTAL DATA FOR $MAPbI_3$

Three dimensional presentations of the electron and hole g-factor tensors measured by TRKR on the $MAPbI_3$ crystal are given in Fig. S5.

The SFRS measured on the $MAPbI_3$ crystal is shown in Fig. S6a. Similar to the $FA_{0.9}Cs_{0.1}PbBr_{0.2}I_{2.8}$ crystal in Fig. ??a, three Raman lines can be well resolved. They correspond to the hole (h), electron (e) and combined (e+h) spin-flip processes. The magnetic field dependences of their Raman shifts are given in Fig. S6b.

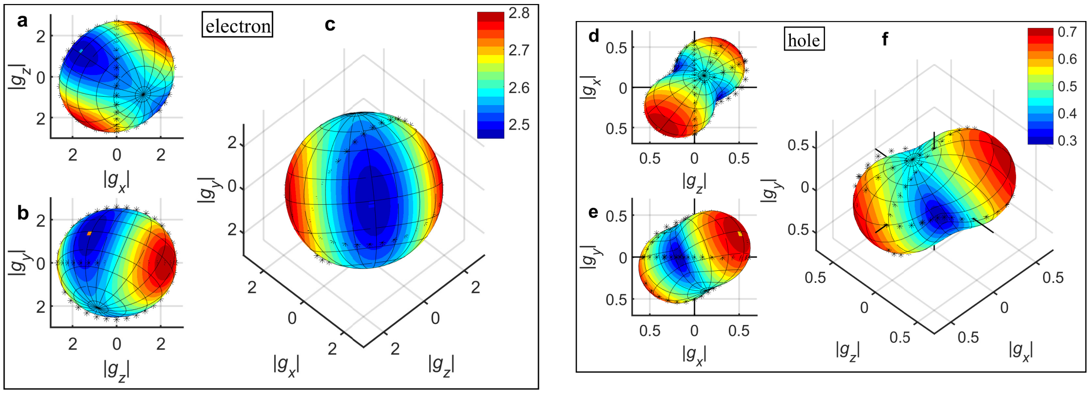

Figure S5: **g-factor tensors of the $MAPbI_3$ crystal measured by TRKR at $T = 7$ K. a-c**, Electron g-factor tensor. **d-f**, Hole g-factor tensor. Stars are experimentally measured data points.

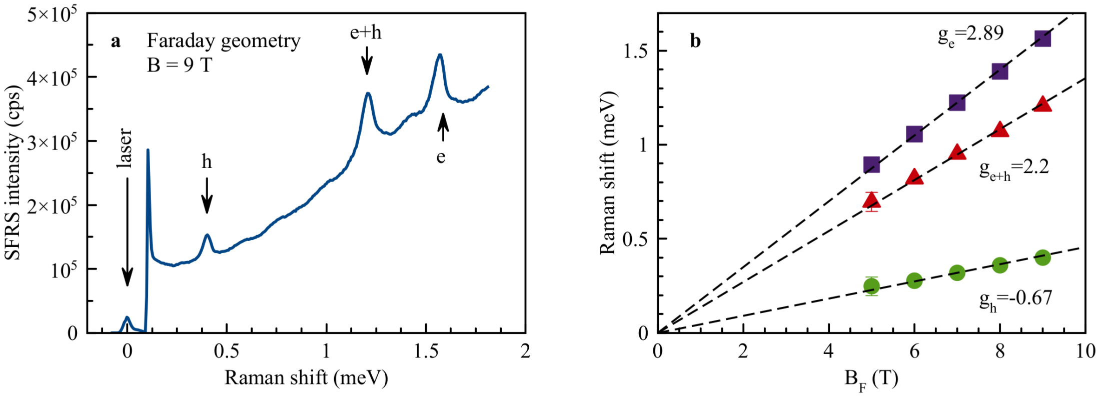

Figure S6: **Spin-flip Raman scattering on the $MAPbI_3$ crystal. a**, SFRS spectrum measured in Faraday geometry at $B = 9$ T and $T = 1.6$ K. Excitation/detection polarization is σ^+/σ^-. **b**, Magnetic field dependence of the Raman shifts of the hole (green), electron (purple) and combined e+h (red) symbols. The experimental data are shown by symbols, the lines are linear fits.

S3. X-RAY CHARACTERIZATION

The crystallographic structure of the investigated samples was characterized using various X-ray-based methods. Mainly, powder and single crystal X-ray diffraction (XRD) measurements were performed for all materials at room temperature. The amount of results of these measurements exceed the limits of the current studies and we want to refer to the publications [S21] for $FA_{0.9}Cs_{0.1}PbBr_{0.2}I_{2.8}$, [S22] for $MAPbI_3$, [S23] for $CsPbBr_3$, [S24] for $MAPbBr_{1.5}I_{1.5}$ and $MAPb(Br_{0.05}I_{0.95})_3$ single crystals.

However, the carrier g-factor anisotropy for $MAPbI_3$ single crystals demands a deep analysis. We have explicitly repeated the results of Ref. [S22], in terms of the performed rocking scan for the used sample. The rocking scan is a method which allows one to quantify the quality of the sample crystal structure. In essence it is an XRD measurement whereby the detector position is fixed at the position of a Bragg reflex and the sample is rotated instead. For a perfect single crystal this results in a sharp peak, while any deviation from the single crystal structure will lead to a broadening. The experiment was performed with a Cu(copper)-Kα1 radiation source providing radiation with a wavelength of $\lambda = 1.5406$ Å. The result of the rocking curve scan is shown in Fig. S7. The full width of half maximum of 0.01596° (57.46 arc sec) was evaluated by fit. This values reflects the high $MAPbI_3$ crystal quality, as compared to the ten times broader (0.16°) peak obtained for $CsPbBr_3$ single crystals grown with the electronic dynamic gradient (EDG) method in Ref. [S26] and that is on the same order of magnitude as the 0.0096° width for $MAPbBr_3$ crystals grown using the in liquid diffused separation induced crystallization (LDSC) method Ref. [S25].

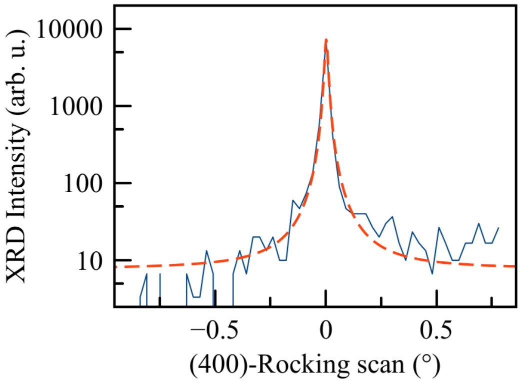

Figure S7: **Rocking scan.** Performed along (400)-Bragg reflex for the $MAPbI_3$ sample at room temperature, with a used radiation source Cu-Kα1. Blue line data and red dashed line fit.

[S1] L. M. Roth, B. Lax, and S. Zwerdling, Theory of optical magneto-absorption effects in semiconductors, Phys. Rev. **114**, 90 (1959)
.
[S2] G. L. Bir and G. E. Pikus, *Symmetry and Strain-induced Effects in Semiconductors* (Wiley/Halsted Press, 1974).
[S3] E. L. Ivchenko, *Optical Spectroscopy of Semiconductor Nanostructures* (Alpha Science Int., Harrow, UK, 2005).
[S4] G. Wang, L. Bouet, M. M. Glazov, T. Amand, E. L. Ivchenko, E. Palleau, X. Marie, and B. Urbaszek, Magneto-optics in transition metal diselenide monolayers, 2D Materials **2**, 034002 (2015).
[S5] C. Hermann and C. Weisbuch, Chapter on "Optical detection of conduction electron spin resonance in semiconductors and its application to $\boldsymbol{k}\cdot\boldsymbol{p}$ perturbation theory" in *Optical Orientation* (North Holland, 1984).
[S6] M. O. Nestoklon, Tight-binding description of inorganic lead halide perovskites in cubic phase, Computational Materials Science **196**, 110535 (2021).
[S7] J. Even, Pedestrian guide to symmetry properties of the reference cubic structure of 3D all-inorganic and hybrid perovskites, J. Phys. Chem. Lett. **6**, 2238 (2015).
[S8] P. Blaha, K. Schwarz, F. Tran, R. Laskowski, G. K. H. Madsen, and L. D. Marks, WIEN2k: An APW+lo program for calculating the properties of solids, J. Chem. Phys. **152**, 074101 (2020).
[S9] F. Tran and P. Blaha, Accurate band gaps of semiconductors and insulators with a semilocal exchange-correlation potential, Phys. Rev. Lett. **102**, 226401 (2009).
[S10] R. A. Jishi, O. B. Ta, and A. A. Sharif, Modeling of lead halide perovskites for photovoltaic applications, J. Phys. Chem. C **118**, 28344 (2014).

[S11] M. Yuan, L. Yuan, Z. Hu, Z. Yu, H. Li, E. M. Barea, J. Bisquert, and X. Meng, In situ spectroscopic ellipsometry for thermochromic $CsPbI_3$ phase evolution portfolio, J. Phys. Chem. C **124**, 8008 (2020).
[S12] M. Cardona, Renormalization of the optical response of semiconductors by electron-phonon interaction, Phys. Stat Sol. (a) **188**, 1209 (2001).
[S13] J. Wiktor, U. Rothlisberger, and A. Pasquarello, Predictive determination of band gaps of inorganic halide perovskites, J. Phys. Chem. Lett. **8**, 5507 (2017).
[S14] J.-M. Jancu, R. Scholz, F. Beltram, and F. Bassani, Empirical spds* tight-binding calculation for cubic semiconductors: General method and material parameters, Phys. Rev. B **57**, 6493 (1998).
[S15] Y. M. Niquet, D. Rideau, C. Tavernier, H. Jaouen, and X. Blase, Onsite matrix elements of the tight-binding Hamiltonian of a strained crystal: Application to silicon, germanium, and their alloys, Phys. Rev. B **79**, 245201 (2009).
[S16] Z. G. Yu, Effective-mass model and magneto-optical properties in hybrid perovskites, Scientific Reports **6**, 28576 (2016).
[S17] P. C. Sercel, J. L. Lyons, D. Wickramaratne, R. Vaxenburg, N. Bernstein, and A. L. Efros, Exciton fine structure in perovskite nanocrystals, Nano Lett. **19**, 4068 (2019).
[S18] P. C. Sercel, J. L. Lyons, N. Bernstein, and A. L. Efros, Quasicubic model for metal halide perovskite nanocrystals, J. Chem. Phys. **151**, 234106 (2019).
[S19] J. Even, L. Pedesseau, M.-A. Dupertuis, J.-M. Jancu, and C. Katan, Electronic model for self-assembled hybrid organic/perovskite semiconductors: Reverse band edge electronic states ordering and spin-orbit coupling, Phys. Rev. B **86**, 205301 (2012).
[S20] M. Graf and P. Vogl, Electromagnetic fields and dielectric response in empirical tight-binding theory, Phys. Rev. B **51**, 4940 (1995).
[S21] Nazarenko, O., Yakunin, S., Morad, V., Cherniukh, I. & Kovalenko, M. V. Single crystals of caesium formamidinium lead halide perovskites: solution growth and gamma dosimetry. *NPG Asia Materials* **9**, e373 (2017).
[S22] Höcker, J., Brust, F., Armer, M. & Dyakonov, V. A temperature-reduced method for the rapid growth of hybrid perovskite single crystals with primary alcohols. *Cryst. Eng. Comm.* **23**, 2202–2207 (2021).
[S23] Dirin, D. N., Cherniukh, I., Yakunin, S., Shynkarenko, Y. & Kovalenko, M. V. Solution-grown $CsPbBr_3$ perovskite single crystals for photon detection. *Chemistry of Materials* **28**, 8470–8474 (2016).
[S24] Dirin, D. N. *unpublished - available on request.* (2022).
[S25] Yao, F., Peng, J., Li, R., Li, W., Gui, P., Li, B., Liu, C., Tao, C., Lin, Q. & Fang, G. Room-temperature liquid diffused separation induced crystallization for high-quality perovskite single crystals. *Nat. Comm.* **11** 1194 (2020).
[S26] Zhang, M., Zheng, Z., Fu, Q., Chen, Z., He, J., Zhang, S., Yan, L., Hu, Y. & Luo, W. Growth and characterization of all-inorganic lead halide perovskite semiconductor $CsPbBr_3$ single crystals. *CrystEngComm* **19** 6797–6803 (2017).
